# Letter to the Editor in response to ‘Parental attachment and depressive symptoms in pregnancies complicated by twin-twin transfusion syndrome: a cohort study’

**DOI:** 10.1186/s12884-021-03688-7

**Published:** 2021-03-22

**Authors:** Georges Rameh, Pia Tohme, Rudy Abi-Habib, Gihad E Chalouhi

**Affiliations:** 1 Department of Obstetrics and Gynecology, American University of Beirut Medical Center, American University of Beirut, Beirut, Lebanon; 2 School of Arts and Sciences, Department of Social Sciences, Lebanese American University, Beirut, Lebanon

**Keywords:** Twin-twin transfusion syndrome, Parental attachment, Parental depression

## Abstract

Twin-twin transfusion syndrome is a highly morbid condition that can affect parental attachment and depression risk. Studies addressing this rare condition are hard to conduct and thus lacking in the literature. In this letter to the editor, we acknowledge the article of Mackie et al. entitled “Parental attachment and depressive symptoms in pregnancies complicated by twin-twin transfusion syndrome: a cohort study” to be of high importance and impact, but would like to discuss the extent of its conclusions, and push towards bigger studies in this field.

## Main text

Twin-twin transfusion syndrome (TTTS) is a highly morbid condition due to vascular anastomoses and imbalanced blood flow between two monochorionic twins, leading to hemodynamic and amniotic fluid discordance [1]. Pregnancies complicated by TTTS have different psychodynamic processes that expecting mothers and fathers have to face throughout the course of pregnancy.

We read with great interest the study by Mackie et al., who published the first UK cohort that studied attachment and depression in parents of pregnancies complicated by TTTS [2]. Their results suggest that parento-fetal attachment increases over the course of pregnancy in mothers, but not fathers. They also state that mothers had more depressive symptoms antenatally compared to fathers but not postnatally.

To our knowledge, this study is the first to include fathers in the analysis of attachment and depression in monochorionic twins undergoing fetoscopic laser ablation (FLA), and it is one of the few studies evaluating the psychological aspect of these procedures. TTTS is an exceptional condition and authors conducting similar studies face many difficulties to get a good sample size and a low loss to follow-up rate.

Previous studies tackling this subject showed that TTTS pregnancy was linked to higher incidence of depressive symptoms and anxiety during and after pregnancy, regardless of the outcome [3].

The UK cohort included 27 couples that were asked to complete attachment and depression scales on the day before FLA treatment, 4 weeks after the ablation and 6–10 weeks postnatally. To note that 26 out of the 54 women presenting for FLA were excluded (48 %), mainly because the partner was not present during the appointment or because they had insufficient time before the procedure to complete the questionnaire [2]. Although this study’s structure and methods are very coherent, it is difficult to acknowledge its results because of three major limitations we wanted to discuss in this letter.

First, having a small sample size with a low response rate is a major weakness, and an extension of the recruitment period to more than 19 months would have improved the power of this study. Excluding couples when the father was not present was important for this paper in order to include both parents in the analysis, but this exclusion criterion affected significantly the sample size and lead to a possible selection bias, since fathers present during the procedure might have higher attachment scores compared to those who were absent. In addition, despite a 100 % agreement to participate of couples approached, only 8 and 5 out of the 27 couples completed the post-ablation and the postnatal questionnaires respectively, and this high loss to follow-up rate might lead to selection bias if women who did not complete the second and third questionnaires were more likely to be depressive. An analysis of “pre-FLA” depressive scores in the families who dropped out might hence provide a stronger argument and shed more light on the possible high drop-out rates in this study. Moreover, having only 8 and 5 couples completing the post-ablation and the postnatal questionnaires does not allow significant conclusions.

Second, baseline anxiety levels should be assessed in similar studies since anxiety might affect parental pre- and post-natal bonding scores as well as depression, especially since TTTS announcement is a stressful and traumatic event and is associated with a significant risk of post-traumatic stress disorder (PTSD) and depression, as well as high levels of anxiety [4]. Supporting this need, a review published by Alhusen et al. reviewing factors that affect maternal-fetal attachment showed that higher anxiety levels are linked to lower bonding scores [5]. Furthermore, anxiety disorders appeared also to show comorbidity with depressive symptoms as well as major depressive disorder [6]. It would also be of interest to assess participants’ coping styles in response to stressful situations given that Mackie et al. argued that mothers might decrease their attachment to their unborn child in risky pregnancies as a protective mechanism. It would be necessary to assess parents’ own attachment style, romantic attachment to partner, or the way they tend to cope with stressful situations, all of which affecting parental bonding, before arguing for parental defensive styles as a coping mechanism [7, 8]. In fact, Mikulincer and Florian discussed parents’ own attachment styles as most influential on prenatal bonding, distancing strategies and mental health throughout pregnancy [7].

Finally, some of the questionnaires used in this study were not validated and the applicability of such questionnaires to a specific high-risk population is questionable. The availability of validated questionnaires in this field is limited but this step is of significant importance in order to compare the results to the normal variant found in such populations.

In conclusion, health care providers have to take into consideration parental attachment styles and depression risks linked to TTTS and its management and should implement screening strategies for both expecting mothers and fathers as well as systematic psychological support. These support groups should target different factors for mothers and fathers as studies converge in finding different predictors of prenatal and postnatal bonding in each parent [8, 9]. This is in line with Mackie et al.’s suggestion of parental gender differences in the type of defensive and protective mechanisms at play when facing stressful situations. The study by Mackie et al. asks very important questions to which all TTTS referral centers need answers to improve patient management. Multicenter and larger sample size studies have to be performed to answer these queries with higher levels of evidence.

## Data Availability

All data generated or analyzed during this study are included in this published article.

## Abbreviations

TTTS: Twin-twin transfusion syndrome
FLA: Fetoscopic laser ablation
PTSD: Post-traumatic stress disorder
